# Reconstruction and analysis of a carbon-core metabolic network for Dunaliella salina

**DOI:** 10.1186/s12859-019-3325-0

**Published:** 2020-01-02

**Authors:** Melanie Fachet, Carina Witte, Robert J. Flassig, Liisa K. Rihko-Struckmann, Zaid McKie-Krisberg, Jürgen E. W. Polle, Kai Sundmacher

**Affiliations:** 1 Max Planck Institute for Dynamics of Complex Technical Systems, Process Systems Engineering, Sandtorstr. 1, Magdeburg, 39106 Germany; 2 Brandenburg University of Applied Sciences, Department of Engineering, Magdeburger Str. 50, Brandenburg an der Havel, 14770 Germany; 3 Brooklyn College of the City University of New York, Department of Biology, 2900 Bedford Avenue, New York, NY 11210 USA; 4 Otto von Guericke University Magdeburg, Process Systems Engineering, Universitätsplatz 2, Magdeburg, 39106 Germany

**Keywords:** *Dunaliella salina*, Metabolic network reconstruction, Central carbon metabolism, Flux balance analysis

## Abstract

**Background:**

The green microalga *Dunaliella salina* accumulates a high proportion of *β*-carotene during abiotic stress conditions. To better understand the intracellular flux distribution leading to carotenoid accumulation, this work aimed at reconstructing a carbon core metabolic network for *D. salina* CCAP 19/18 based on the recently published nuclear genome and its validation with experimental observations and literature data.

**Results:**

The reconstruction resulted in a network model with 221 reactions and 212 metabolites within three compartments: cytosol, chloroplast and mitochondrion. The network was implemented in the MATLAB toolbox CellNetAnalyzer and checked for feasibility. Furthermore, a flux balance analysis was carried out for different light and nutrient uptake rates. The comparison of the experimental knowledge with the model prediction revealed that the results of the stoichiometric network analysis are plausible and in good agreement with the observed behavior. Accordingly, our model provides an excellent tool for investigating the carbon core metabolism of *D. salina*.

**Conclusions:**

The reconstructed metabolic network of *D. salina* presented in this work is able to predict the biological behavior under light and nutrient stress and will lead to an improved process understanding for the optimized production of high-value products in microalgae.

## Introduction

Microalgae received increased attention over recent years due to their ability to produce high-value compounds such as polyunsaturated fatty acids and carotenoids [1–3]. Optimizing microalgal growth and product compositions in order to facilitate economically feasible mass production is still challenging. A better understanding of the complex algal metabolism is an important prerequisite overcoming this hurdle. In regards to algal metabolism, the halophilic unicellular green alga *Dunaliella salina* is an excellent model organism to investigate changes in metabolism [4] as the physiology of the switch from primary growth to secondary stress metabolism with glycerol and carotenoid accumulation is very well known [5–7]. In addition, *D. salina* remains one of the few microalgae currently being commercialized for *β*-carotene production on a large scale [8].

The construction of dynamic-kinetic growth models using ordinary differential equations (ODEs) is a well-established formalism in bioprocess engineering. These models allow for prediction of biomass growth, nutrient uptake and metabolite production and enable the identification of bottlenecks in the process setup for both lab-scale and large-scale outdoor cultivation systems [9–11]. Simplified growth models are robust and computationally inexpensive. However, they might be only valid for a certain range of environmental conditions, thus limiting predictive capabilities for extrapolation outside the experimental region [12].

It is known that metabolic processes are based on complex reaction pathways throughout different subcellular compartments and its integration into a metabolic model is a prerequisite to get insight into the formation and regulation of metabolites [13]. Several flux-balance models of different plant and algal species have already been published. These include models for higher plants *Arabidopsis* [14], barley [15], *Brassica napus* seeds [16] and green microalgae such as *Chlamydomonas* [17–21], *Chlorella* [22–26] and *Ostreococcus* [27].

Currently, the productivities of microalgae are still below their actual potential. However, metabolic network reconstructions are the basis for stoichiometric modeling efforts and they have the ability to provide theoretical maximal substrate and product yields as well as calculation of internal metabolic rates. Furthermore, they enable in silico identification of genetic intervention strategies that guarantee a specified product yield, e.g. by engineering of the carotenoid or lipid synthesis pathways [28]. Usually, methods such as flux balance analysis (FBA) are used to determine the steady-state flux distribution in a metabolic network under given input conditions by maximization of an objective function. Moreover, extensions for FBA methods such as dynamic flux balance analysis (DFBA) exist, accounting for unbalanced growth conditions and dynamic extracellular effects on intracellular metabolism [21, 29]. This enables exploration of metabolic flux distributions consistent with stoichiometric and thermodynamic constraints as well as constraints formulated according to experimental data [30].

Since *D. salina* is the richest known source of natural *β*-carotene, a metabolic network model is highly beneficial to fully exploit the biotechnological potential of this alga. So far, for *D. salina* some metabolic profiling information is available [31, 32], and the first growth models have recently been created [11, 33, 34]. In addition, the genome of *D. salina* has been released (http://genome.jgi.doe.gov/DunsalCCAP1918/DunsalCCAP1918.info.html) [35]. However, the annotation of the nuclear genome is challenging since it contains a high number of long introns and extensive repeats, complicating proper gene model construction. Therefore, a genome scale metabolic reconstruction for the industrially relevant microalga *D. salina* is still missing. Based on the nuclear genome of strain CCAP19/18 [35], a manual reconstruction of a carbon-core metabolic network was performed. The aim of the reconstructed stoichiometric network is to describe the metabolic flux distribution leading to the accumulation of the major biomass constituents in *D. salina* under fluctuating light and nutrient conditions.

## Results

### Reconstruction of a stoichiometric network for the carbon-core metabolism

By linking the annotated genetic information from [35] with bioinformatic knowledge from databases (e.g. KEGG, Kyoto Encyclopedia of Genes and Genomes), a stoichiometric network for the carbon-core metabolism with interfaces to the amino acid metabolism of *D.salina* CCAP19/18 that comprises 221 reactions and 213 metabolites in three different compartments (chloroplast, cytosol and mitochondrion) was reconstructed. A comprehensive list of reactions and compounds in the metabolic network can be found in the Additional file 1. All entries in the list of reactions carrying an EC number (Enzyme commission number) and KEGG ID are annotated enzymes of the *D.salina* genome. Although more extensive metabolic networks exist for a variety of unicellular algae [20, 36, 37], the purpose of our work was to create the first reduced network that would still be capable of predicting biomass composition and productivities.

**Fig. 1:**
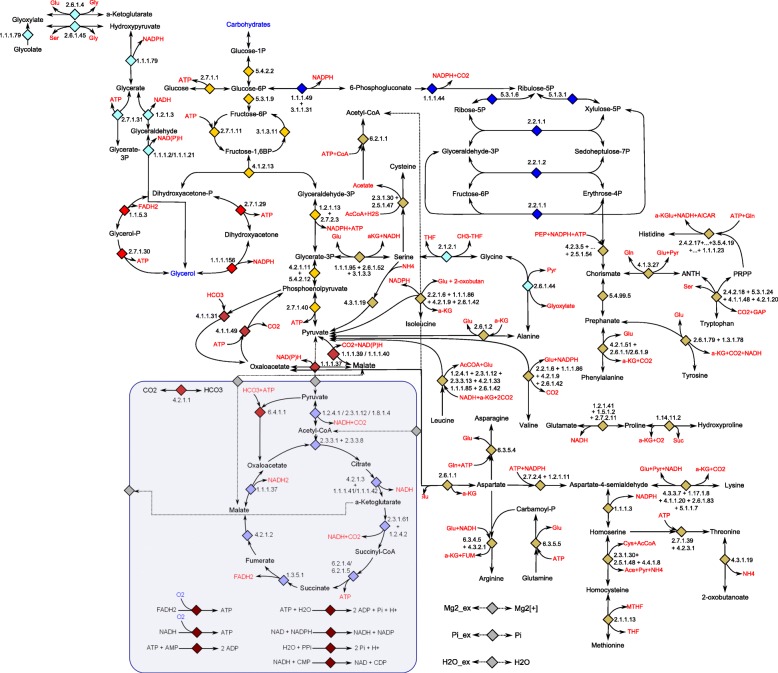
Network map of carbon core metabolism in the cytosol and mitochondrion. For reasons of simplicity linear reactions were merged. The arrows display the direction and reversibility of the reactions. The blue font color refers to metabolites modeled as biomass compounds and the red font color refers to key reaction components such as energy and reduction equivalents

**Fig. 2:**
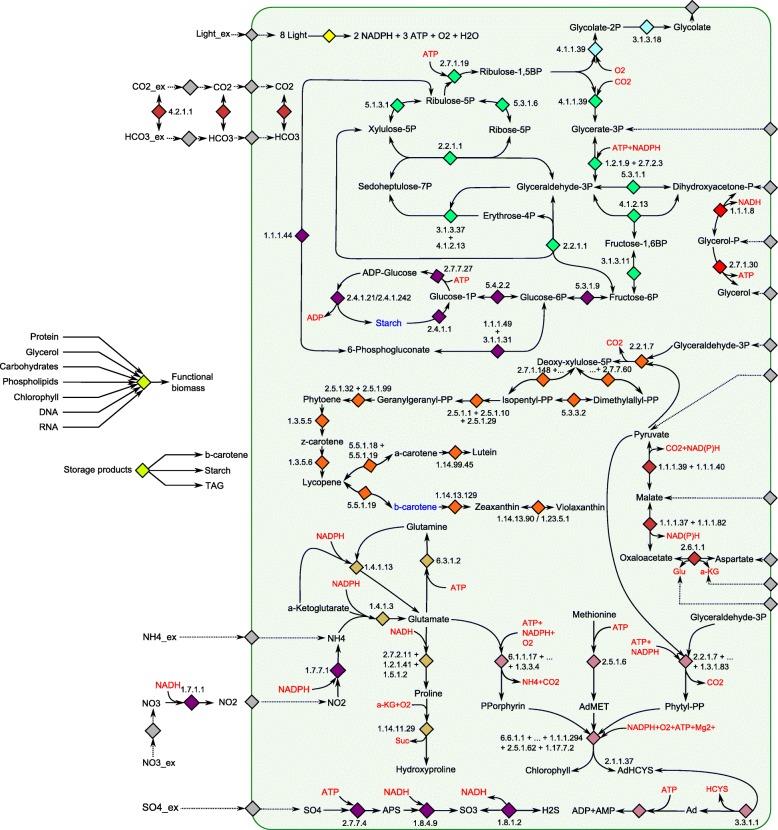
Network map of carbon core metabolism in the chloroplast. For reasons of simplicity linear reactions were merged. The arrows indicate the direction and reversibility of the reactions. The gray boxes indicate shuttling of metabolites between the considered compartments. The blue font color refers to metabolites modeled as biomass compounds and the red font color refers to key reaction components such as energy and reduction equivalents

**Fig. 3:**
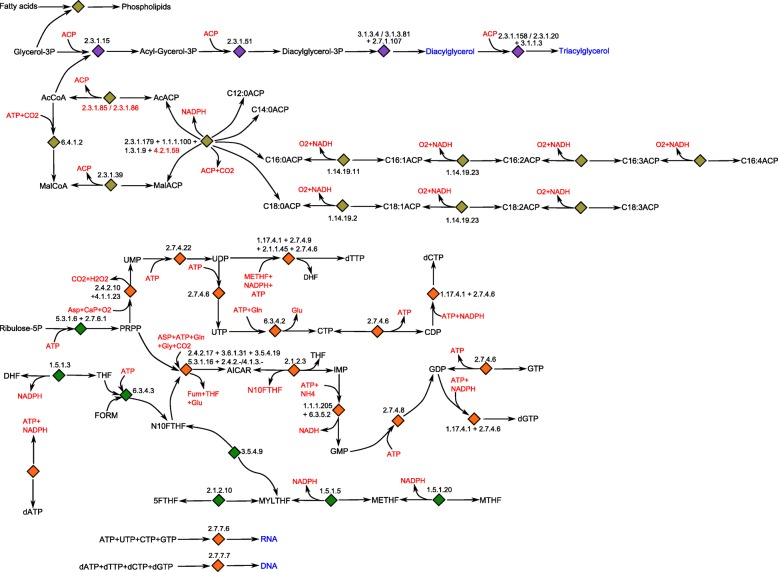
Network map of the fatty acid and nucleic acid metabolism. The arrows indicate the direction and reversibility of the reactions. For reasons of simplicity linear reactions were merged. The gray boxes indicate shuttling of metabolites between the considered compartments. The blue font color refers to metabolites modeled as biomass compounds and the red font color refers to key reaction components such as energy and reduction equivalents

Figure 1, 2 and 3 show the network maps for the cytosol, the chloroplast and the mitochondrion respectively. To create the metabolic map with subcellular localization of enzymes, the prediction program PredAlgo was used. The prediction tool had been developed and designed to determine the subcellular localization of nuclear-encoded enzymes in *C. reinhardtii* [38]. Consequently, PredAlgo distinguishes between the following three compartments: the mitochondrion, the chloroplast, and the cytosol. The study of [38] showed that the application of PredAlgo led to an improved discrimination between plastidal and mitochondrial-localized proteins. As stated by its authors, PredAlgo works most accurately for the genus of *Chlamydomonas* and related green algal species (Chlorophyta).

Algae of the genus *Dunaliella* and *Chlamydomonas* are closely related, because they both belong to the order of Volvocales [39], a comparison of annotated enzymes for the calvin cycle, the carbon-core metabolism and the isoprenoid biosynthesis of *D.salina* and *C. reinhardtii* showed a high degree of similarity [40]. In addition, there is a broad consensus that the carbon core metabolisms of green microalgae are conserved along several lineages since almost 90% of the functional annotated proteins of *C. reinhardtii* and of other microalgal proteins are homologs of *Arabidopsis thaliana* proteins [41]. For instance, similar to *C. reinhardtii*, the enzyme triose-phosphate isomerase (EC 5.3.1.1) is only present as one gene within the genome of *D. salina*. PredAlgo predicted a chloroplast localization, thus confirming the expected localization with the Calvin-Benson-Bassham cycle for carbon acquisition in the plastid of photosynthetic organisms. Moreover, multiple green algal species (Chlorophyta) share the presence of a glycolytic enolase (EC 4.2.1.11) with cytosolic localization rather than a plastid-localized enolase enzyme [42].

A major difference between the model alga *C. reinhardtii* and *D.salina* is the adaptation of *D.salina* to life under high salinities, whereas *C. reinhardtii* exclusively lives in soil and freshwater. Therefore, metabolism of *D.salina* was expected to reveal not only similarities, but also differences in subcellular localization of some of the annotated enzymes. For example, the enzyme the carbonic anhydrase (CA, EC 4.2.1.1) was included in the network to ensure carbon acquisition under high salt conditions. The genome of *C. reinhardtii* contains three *α*-type, six *β*-type and three *γ*-type CAs [43]. In contrast to freshwater species, [44] identified five *α*-type CAs and three *γ*-type CAs, but no *β*-type CAs in *D. salina* CCAP19/18. The newly identified *α*-type CA (DsCA2b) is suggested to improve CO2 assimilation under hypersaline conditions [44]. Based on results of [45], a plasma membrane localization acting on the extracellular side was assumed. Although a variety of genes code for different classes of carbonic anhydrases [44], we only considered the extracellular version in our model, because it is specific to *Dunaliella*.

In contrast, multiple green algal species (Chlorophyta) share the presence of a glycolytic enolase (EC 4.2.1.11) with cytosolic localization rather than a plastid-localized enolase enzyme [42]. The glycerol cycle is initiated by the formation of glycerol-3-phosphate from dihydroxyacetone-phosphate, either provided through glycolytic reactions in the cytosol or through the reductive pentose phosphate pathway in the chloroplast [46]. This reversible reaction is catalyzed by the glyceraldehyde-3-phosphate dehydrogenase (GPDH), which exists as two different enzymes, Nicotinamide-adenine dinucleotide (NAD+)-dependent enzyme (EC 1.1.1.8) with plastidal and cytosolic localization and the ubiquinone-dependent enzyme (EC 1.1.5.3) with cytosolic localization bound to the mitochondrial membrane. The following formation of glycerol from glycerol-3-phosphate was considered to be performed by the glycerol kinase (EC 2.7.1.30). The presented hypothesis of the glycerol cycle within the cytosol also includes the removal of glycerol by conversion to dihydroxyacetone via the dihydroxyacetone reductase (EC 1.1.1.156) and subsequent phosphorylation to dihydroxyacetone-phosphate by the glycerone kinase (EC 2.7.1.29), thus connecting the glycerol cycle back to the glycolysis. Another option for cells to dispose of glycerol may be through general alcohol dehydrogenases (EC 1.1.1.2/1.1.1.21). This is a novel finding, indicating that glycerol could be connected to the carbon core metabolism in more ways than previously proposed, possibly providing a second glycerol cycle in *D. salina*.

Regarding carotenoid biosynthesis, genes coding for all of the enzymes of the plastid localized isoprenoid biosynthesis referred to as the Methyl-Erythritol-Phosphate (MEP) pathway were identified [35]. In addition, genes for all prenyl transferases needed to synthesize phytoene were found in the genome and all genes coding for enzymes required for reactions leading to *β*-carotene were identified.

### Flux balance analysis of low and high-light scenarios under nutrient repletion and depletion

The reconstructed network was implemented in the MATLAB toolbox CellNetAnalyzer and checked for consistency and feasibility by using the function Check feasibility of flux scenario. Additionally, a FBA was carried out to analyze the plausibility of the flux distribution under varying light and nutrient conditions. The input fluxes for light (*Ex*01) and nutrients (*Ex*06) in the FBA scenarios were fixed according to experimentally obtained values for cultivations in a flat-plate bioreactor setup. For the nitrogen uptake rate, a maximal rate of 0.19 mmol/(g dw · h) for the nitrogen-replete scenarios and 0.001 mmol/(g dw · h) for the nitrogen-limited scenarios were calculated. Additionally, the maximal uptake rate for light (*Ex*01) was adapted to 800 mmol/(g dw · h) according to experimental values obtained in flat-plate bioreactor experiments under high light conditions [29]. The maintenance ATP requirement (Reaction R192) was calculated by dynamic modeling from chemostat experiments conducted in a laboratory flat-plate bioreactor and was fixed to 0.92 mmol/(g dw · h).

The results of the FBA for the defined scenarios (A-H) are listed in Tables 1 and 2. In the scenarios A and B, the nitrogen source represented by the metabolite nitrate (NO _3_^−^) was set to the maximal reaction rate of 0.19 mmol/(g dw · h) to simulate autotrophic growth under nitrogen-replete conditions for low and high light conditions. For the scenarios C and D, the nitrate flux (Ex06) was set to 0.001 mmol/(g dw · h) to simulate autotrophic growth under nitrogen-limited conditions. The objective function was defined to maximize biomass growth under autotrophic conditions (represented by the biomass-forming reaction *μ*) and the internal fluxes were calculated.

**Table 1:** Input conditions and predicted growth rates for the defined scenario A-C

Scenario	A	B	C	D
Light	LL	HL	LL	HL
Nutrients	nutrient-replete		nutrient-depleted	
Input conditions:				
Light (Ex01)	320	800	320	800
Nutrients (Ex06, NO _3_^−^)	0.19	0.19	0.001	0.001
Objective function	max(*μ*)			
Calc. growth rate in 1/h	0.1287	0.7934	0.0007	0.0007
Calc. *β*-carotene production in mmol/(gdw ·h)	0	0	0	0

**Table 2:** Input conditions and predicted growth rates for the defined scenario E-H

Scenario	E	F	G	H
Light	LL	HL	LL	HL
Nutrients	nutrient-replete		nutrient-depleted	
Input conditions:				
Light (Ex01)	320	800	320	800
Nutrients (Ex06, NO _3_^−^)	0.19	0.19	0.001	0.001
Objective function	max(*μ*,*Car*14)			
Calc. growth rate in 1/h	0.1287	0.7934	0.0007	0.0007
Calc. *β*-carotene production in mmol/(g dw ·h)	0.6962	1.2972	0.7556	1.5359

The simulations for the scenarios E - G were carried out under the same nitrogen-replete and depleted conditions as A - D with the only difference that the maximization of the *β*-carotene flux (Car14) was added to the objective function to test whether the flux distribution enables a growth-coupled accumulation of secondary pigments. The objective function for these scenarios is defined as follows: maximization of biomass growth (reaction *μ*) and *β*-carotene production (reaction Car14).

The resulting growth rates *μ* for the biomass-maximizing scenarios A - D revealed a nitrogen limited growth regime. Under nitrogen-replete conditions, growth rates of 0.1287 h^-1^ and 0.7934 h^-1^ were predicted for the low light and high-light input flux (Ex01). The predicted growth rate under low-light conditions (3.09 d^-1^) is only slightly higher than previously published growth data for *D. salina* CCAP19/18 where a maximal growth rate of 1.71 d^-1^ was predicted by dynamic-kinetic modeling of batch cultivation data [47]. In the nutrient-depleted scenarios C and D, no biomass growth (*μ*=0.0007 h^-1^) occured neither under low light nor high light conditions.

In scenario A - D, biomass production occurred without any formation of *β*-carotene as a side product, meaning that the *β*-carotene flux Car14 is always 0 mmol/(g dw · h) (Table 1). Since the objective function did only include the biomass growth (*μ*) under nitrogen-replete conditions it is biologically plausible that *β*-carotene formation was suppressed in the flux scenarios A - D. As described by [6] and [11] oversaturating light conditions and nutrient repletion led only to moderate *β*-carotene accumulation, whereas oversaturating light combined with nutrient stress is the most potent inducer of secondary carotenoids in *D. salina*.

The tested scenarios E - H (Table 2) were similar to A - D despite the extension of the objective function to maximize the *β*-carotene flux (Car14). The same growth rates as in scenarios A - D were calculated (0.1287 h^-1^ and 0.7934 h^-1^ for nutrient-replete conditions and 0.0007 h^-1^ for nutrient-depleted conditions). However, the predicted *β*-carotene flux was different compared to scenarios A - D.

For the nutrient-replete scenarios E and F, the lowest *β*-carotene accumulation of 0.6962 mmol/(g dw · h) was predicted under low light conditions whereas a *β*-carotene flux (Car14) of 1.2972 mmol/(g dw · h) was predicted under high light conditions. Under nutrient-depleted conditions, the predicted *β*-carotene flux (Car14) was 0.7556 mmol/(g dw · h) under low light and 1.5359 mmol/(g dw · h) under high light conditions (Table 2).

## Discussion

The reconstruction of a stoichiometric network for the carbon-core metabolism of *D.salina* CCAP19/18 was performed from annotated genetic information of with knowledge from bioinformatic databases such as KEGG. The size of the metabolic network for *D.salina* (221 reactions and 213 metabolites in three different compartments: chloroplast, cytosol and mitochondrion) is in the range of previously published reduced networks for green microalgae (e.g. for *C.reinhardtii* with 160 reactions, 164 metabolites in two compartments by [48] or with 259 reactions, 267 metabolites in 6 compartments by [49]).

With respect to the carotenoid synthesis, it was essential that all enzymes of the isoprenoid biosynthesis were identified, because under environmental stress cells of *D. salina* de-novo synthezise up to 10% of their dry weight as the isoprenoid molecule *β*-carotene [5]. Furthermore, the sequencing of various green algal species was an important prerequisite to study their different accumulation patterns of TAGs and carotenoids. [50] proposed that the pattern of carbon flow towards TAG or carotenoids is regulated by the NAD(P)H reduction state and the presence of bypass mechanisms such as pyruvate dehydrogenase (PDH). In the case of *D. salina*, the downregulation of PDH induced by high NAD(P)H levels under abiotic stress conditions favors *β*-carotene hyperaccumulation rather than massive TAG accumulation [50].

The results of the predicted *β*-carotene fluxes shown in Table 2 are supported by experimental observations for bioreactor cultivations of *D. salina* CCAP19/18 where low light and nutrient depletion led to the lowest *β*-carotene fraction of 30 mg/g dw followed by high light without nutrient stress with 43 mg/g dw. The highest experimentally observed *β*-carotene fraction was detected under high light coupled with nutrient stress, namely 80 mg/g dw [47]. This is in line with the biological function of *β*-carotene acting as a metabolic sink under conditions where growth is limited by excess light or nutrient stress [51]. The absence of biomass production in scenarios C-D and G-H is plausible, since nitrogen depletion leads to inhibition of protein biosynthesis which is a prerequiste for growth.

## Conclusion

This work presents a metabolic network reconstruction of the carbon-core metabolism of *D.salina* CCAP19/18 based on the recently announced annotated genome [35]. The network comprises 221 reactions with 212 metabolites in three compartments (chloroplast, cytsol and mitochondrion). The network was implemented in the MATLAB toolbox CellNetAnalyzer and a flux balance analysis was carried out under various light and nutrient scenarios. The simulation results were compared with experimental observations of *D.salina* cultivated under nutrient repletion and depletion in a flat-plate photobioreactor [47]. All model predictions could be confirmed by experimental data and biological knowledge of *D.salina* metabolism. In conclusion, the metabolic network reconstruction is suitable to gain a better understanding of the flux distribution in the carbon core metabolism during carotenogenesis in *D. salina*. The ongoing experimental and computational advances will thereby accelerate the engineering of industrially valuable strains and provides the basis for effective biotechnology with photosynthetic microorganisms.

## Methods

### Reconstruction of the stoichiometric network

The stoichiometric model of *D. salina* CCAP19/18 carbon-core metabolism was reconstructed using a traditional (bottom-up) approach, which relied on manual reconstruction. It is based on the assignment of all annotated genes in the nuclear genome of D. salina CCAP19/18 to their proteins and the corresponding reactions supported by biological databases such as KEGG [35]. The complete reaction list is given in the Additional file 2. The graphical representation of the network was created in the vector graphics editor Inkscape (Version 0.92), which is based on [48].

Some metabolites in our stoichiometric network model may have one or more designations denoting their presence in different cellular compartments. Exchange reactions were added allowing the import and export between the considered cellular compartments.

### Implementation and validation of the network

The complete set of reaction equations was implemented in the MATLAB toolbox CellNetAnalyzer and checked for feasibility [52]. Unless otherwise stated (e.g. for the nutrient uptake flux or the light flux) the lower and upper bounds for irreversible reactions were fixed to 0 - 100 mmol/(g dw · h), whereas reversible reaction bounds were fixed to -100 - 100 mmol/(g dw · h). The maximum flux boundaries of 100 mmol/(g dw · h) rely on biologically realistic values and are commonly used in FBA. For example, [53] categorized fluxes as low (<5 mmol/(g dw · h)), medium, (>5–10 mmol/(g dw · h)), and high (>10 mmol/(g dw · h)). The FBA was carried out for different objective functions as well as light and nutrient uptake rates by using the function Flux optimization. The network implementation and the values for the flux scenarios are provided in the Additional file 3.

## Supplementary information

**Additional file 1** List of reactions, metabolites and biomass composition.

**Additional file 2** SBML file.

**Additional file 3** Flux values, constraints, gene-protein reaction associations.

## Data Availability

The datasets used and/or analyzed during the current study are provided in the Supplementary Material.

## Abbreviations

CA: Carbonic anhydrase
CCAP: Culture Collection of Algae and Protozoa
DFBA: Dynamic Flux Balance Analysis
EC: Enzyme commission
FBA: Flux Balance Analysis
KEGG: Kyoto Encyclopedia of Genes and Genomes MEP: Methyl-Erythritol-Phosphate
ODE: Ordinary Differential Equation
