# Supplementary material for: Reconstruction and analysis of a carbon-core metabolic network for Dunaliella salina
Source: BMC Bioinformatics. 2020 Jan 2;21:1. doi: 10.1186/s12859-019-3325-0 (PMC6941287; doi:10.1186/s12859-019-3325-0)
Supplement: Supplementary file 1 — Additional file 1 List of reactions, metabolites and biomass composition. [file 12859_2019_3325_MOESM1_ESM.pdf]

Metabolic network information

1 Reaction equations

No.	ID	Reaction	E.C.	KEGG ID
R001	Light1	8 Light + 3 ADP + 3 Pi + H[+] + 2 cNADP → O2 + H2O + 2 cNADPH + 3 ATP		
R002	Cal01	cCO2 + H2O + cRu15DP → 2 c3PG + 2H[+]	4.1.1.39	R00024
R003	Cal02	ATP + NADPH + c3PG ↔ ADP + NADP + H2O + cGAP	2.7.2.3	R01512
			1.2.1.9	R01058
R004	Cal03	cGAP ↔ cDHAP	5.3.1.1.	R01015
R005	Cal04	cDHAP + cGAP ↔ cF16P	4.1.2.13	R01068
R006	Cal05	H2O + cF16P ↔ Pi + cF6P	3.1.3.11	R00762
R007	Cal06	cF6P + cGAP ↔ cE4P + cX5P	2.2.1.1	R01067
R008	Cal07	H2O + cE4P + cGAP ↔ Pi + cS7P	3.1.3.37	R01845
			4.1.2.13	R01829
R009	Cal08	cGAP + cS7P ↔ cR5P + cX5P	2.2.1.1	R01641
R010	Cal09	cX5P ↔ cRu5P	5.1.3.1	R01529
R011	Cal10	cR5P ↔ cRu5P	5.3.1.6	R01056
R012	Cal11	ATP + cRu5P → ADP + cRu15DP	2.7.1.19	R01523
R013	Gluc01	cG6P + H2O + NADP ↔ c6PG + NADPH + H[+]	1.1.1.49	R00835
			3.1.1.31	R02035
R014	Gluc02	c6PG + NADP ↔ cCO2 + NADPH + cRU5P + H[+]	1.1.1.44	R01528
R015	Gluc03	cF6P ↔ cG6P	5.3.1.9	R00771
R016	Gluc04	cG6P ↔ cG1P	5.4.2.2	R08639
R017	Gluc05	ATP + cG1P ↔ cADP-G + PPi	2.7.7.27	R00948
R018	Gluc06	cADP-G ↔ ADP + Starch	2.4.1.21	R06049
R019	Gluc07	Starch + Pi ↔ cG1P	2.4.1.1	R06185
R020	CAM01	cHCO3[-] + H[+] ↔ cCO2 + H2O	4.2.1.1	R10092
R021	CAM02	HCO3[-] + H[+] ↔ CO2 + H2O	4.2.1.1	R10092
R022	CAM03	mHCO3[-] + H[+] ↔ mCO2 + H2O	4.2.1.1	R10092
R023	CAM04	cMAL + NAD ↔ cPYR + CO2 + NADH	1.1.1.39	R00214
R024	CAM05	cMAL + NADP ↔ cPYR + CO2 + NADPH	1.1.1.40	R00216
R025	CAM06	cMAL + NAD ↔ cOXA + NADH + H[+]	1.1.1.37	R00342
R026	CAM07	cMAL + NADP ↔ cOXA + NADPH + H[+]	1.1.1.82	R00343
R027	CAM08	cASP + cAKG ↔ cOXA + cGLU	2.6.1.1	R00355
R028	CAM09	MAL + NAD ↔ PYR + CO2 + NADH	1.1.1.39	R00214
R029	CAM10	MAL + NADP ↔ PYR + CO2 + NADPH	1.1.1.40	R00216
R030	CAM11	MAL + NAD ↔ OXA + NADH + H[+]	1.1.1.37	R00342
R031	CAM12	ATP + OXA → ADP + CO2 + PEP	4.1.1.49	R00341
R032	CAM13	HCO3[-] + PEP → OXA + Pi	4.1.1.31	R00345
R033	CAM14	ATP + mHCO3[-] + mPYR → ADP + mOXA + Pi	6.4.1.1	R00344
R034	Pres01	cRu15DP + O2 → c3PG + cGLYCOL2P	4.1.1.39	R03140
R035	Pres02	cGLYCOL2P + H2O → cGLYCOL + Pi	3.1.3.18	R01334
R036	Pres03	GLYCOL + NAD ↔ GLYOX + NADH + H[+]	1.1.1.79	R00465
R037	Pres04	SER + GLYOX ↔ GLY + HydPyr	2.6.1.45	R00588
R038	Pres05	GLU + GLYOX ↔ GLY + AKG	2.6.1.4	R00372
R039	Pres06	H[+] + HydPyr + NADH ↔ GLYCA + NAD	1.1.1.79	R01392
R040	Pres07	ATP + GLYCA ↔ ADP + 3PG	2.7.1.31	R01514
R041	Pres08	GA + NAD + H2O ↔ GLYCA + NADH + H[+]	1.2.1.3	R01752
R042	Pres09	GLYC + NAD ↔ GA + NADH + H[+]	1.1.1.21	R01036
R043	Pres10	GLYC + NADP ↔ GA + NADPH + H[+]	1.1.1.2	R01041
R044	Pres11	GLY + H2O + METHF ↔ SER + THF	2.1.2.1	R00945
R045	Pres12	GLY + NAD + THF ↔ CO2 + METHF + NADH + NH4[+]	1.4.4.2	R01221
R046	Pres13	GLY + H[+] + PYR ↔ ALA + GLYOX	2.6.1.44	R00369

R047	Gly01	G6P ↔ G1P	5.4.2.2	R08639
R048	Gly02	F6P ↔ G6P	5.3.1.9	R00771
R049	Gly03	ATP + F6P → ADP + F16P	2.7.1.11	R00756
R050	Gly04	F16P + H2O → F6P + Pi	3.1.3.11	R00762
R051	Gly05	DHAP + GAP ↔ F16P	4.1.2.13	R01068
R052	Gly06	GAP + NADP + Pi + ADP ↔ 3PG + ATP + H[+] + NADPH	1.2.1.13	R01063
			2.7.2.3	R01512
R053	Gly07	3PG ↔ H2O + PEP	5.4.2.1	R01518
			4.2.1.11	R00658
R054	Gly08	ADP + PEP ↔ ATP + PYR	2.7.1.40	R00200
R055	Gly09	ATP + GLUC ↔ ADP + G6P	2.7.1.1	R00299
R056	Gly10	G1P ↔ CARB + Pi		
R057	PP01	G6P + H2O + NADP ↔ 6PG + NADPH + 2 H[+]	1.1.1.49	R00835
			3.1.1.31	R02035
R058	PP02	6PG + NADP ↔ CO2 + NADPH + RU5P + H[+]	1.1.1.44	R01528
R059	PP03	RU5P ↔ R5P	5.3.1.6	R01056
R060	PP04	RU5P ↔ X5P	5.1.3.1	R01529
R061	PP05	R5P + X5P ↔ GAP + S7P	2.2.1.1	R01641
R062	PP06	GAP + S7P ↔ E4P + F6P	2.2.1.2	R08575
R063	PP07	F6P + GAP ↔ E4P + X5P	2.2.1.1	R01067
R064	TCA01	CoA + NAD + mPYR → mAcCoA + mCO2 + NADH	1.2.4.1	R00209
			2.3.1.9	
			1.8.1.4	
R065	TCA02	mAcCoA + H2O + mOXA → mCIT + CoA + H[+]	2.3.3.1	R00351
			2.3.3.8	R00352
R066	TCA03	mCIT + NAD → mAKG + mCO2 + NADH	1.1.1.41	R00709
			1.1.1.42	R00267
			4.2.1.3	R01324
R067	TCA04	mAKG + CoA + NAD → mCO2 + NADH + mSUCCoA	1.2.4.2	R01700
			2.3.1.61	R02570
R068	TCA05	ADP + Pi + mSUCCoA → ATP + CoA + mSUC	6.2.1.4	R00432
			6.2.1.5	R00405
R069	TCA06	FAD + mSUC → FADH2 + mFUM	1.3.5.1	R02164
R070	TCA07	mFUM + H2O → mMAL	4.2.1.2	R01082
R071	TCA08	NAD + mMAL ↔ NADH2 + mOXA	1.1.1.37	R00342
R072	Glyc01	DHAP + ADP ↔ DHA + ATP	2.7.1.29	R01011
R073	Glyc02	DHA + H[+] + NADPH ↔ GLYC + NADP	1.1.1.156	R01039
			1.1.1.2	R01041
R074	Glyc03	ADP + cGLYC3P → ATP + cGLYC	2.7.1.30	R00847
R075	Glyc04	GLYC3P + FAD ↔ DHAP + FADH2	1.1.5.3	R00848
R076	Glyc05	cGLYC3P + NAD ↔ cDHAP + H[+] + NADH	1.1.1.8	R00842
R077	Glyc06	ADP + GLYC3P → ATP + GLYC	2.7.1.30	R00847
R078	NS01	H[+] + NADH + NO3 → H2O + NAD + NO2	1.7.1.1	R00794
R079	NS02	5 H[+] + 3 NADPH + cNO2 → cNH4[+] + 2 H2O + 3 NADP	1.7.7.1	R00790
R080	NS03	ATP + cSO4 → cAPS + PPi	2.7.7.4	R00529
R081	NS04	cAPS + NADH → AMP + NAD + cSO3	1.8.4.9	R05717
R082	NS05	5 H[+] + 3 NADPH + cSO3 ↔ cH2S + 3 H2O + 3 NADP	1.8.1.2	R00858
R083	AA01	cAKG + H[+] + NADPH + NH4[+] → cGLU + H2O + NADP	1.4.1.3	R00248
R084	AA02	ATP + cGLU + NH4[+] → ADP + cGLN + H[+] + Pi	6.3.1.2	R00253
R085	AA03	cAKG + cGLN + H[+] + NADPH ↔ NADP + 2 cGLU	1.4.1.13	R00114
R086	AA04	3PG + GLU + H2O + NAD ↔	1.1.1.95	R01513
		AKG + H[+] + NADH + Pi + SER	2.6.1.52	R04173
			3.1.3.3	R00582
R087	AA05	SER → NH4[+] + PYR	4.3.1.19	R00220
R088	AA06	AcCoA + H2S + SER ↔ Ace + CYS + CoA + H[+]	2.3.1.30	R00586
			2.5.1.47	R00897

R089	AA07	ATP + Ace + CoA → ADP + AcCoA + Pi	6.2.1.1	R00235
R090	AA08	GLU + PYR → AKG + ALA	2.6.1.2	R00258
R091	AA09	H[+] + THR ↔ 2OXOB + NH4[+]	4.3.1.19	R00996
R092	AA10	2OXOB + GLU + H[+] + NADPH + PYR ↔	2.2.1.6	R08648
		AKG + CO2 + H2O + ILE + NADP	1.1.1.86	R05069
				R04439
			4.2.1.9	R01209
			2.6.1.42	R02199
R093	AA11	2 H[+]+ GLU + NADPH + PYR ↔	2.2.1.6	R00006
		CO2 + H2O + NADP + VAL	1.1.1.86	R03051
			4.2.1.9	R01209
			2.6.1.42	R01214
R094	AA12	2 PYR + AcCoA + GLU + H[+] + NAD + NADPH ↔	1.2.4.1	R00014
		AKG + CoA + LEU + NADH + NADP + 2 CO2		R03270
			2.3.1.9	R02569
			2.3.3.13	R01213
			4.2.1.33	R10170
			1.1.1.85	R10052
			2.6.1.42	R01090
R095	AA13	2 PEP + ATP + E4P + NADPH →	4.2.3.5	R01714
		ADP + CHO + NADP + 4 Pi	2.5.1.19	R03460
			2.7.1.71	R02412
			1.1.1.25	R02413
			4.2.1.10	R03084
			4.2.3.4	R03083
			2.5.1.54	R01826
R096	AA14	CHO ↔ PRE	5.4.99.5	R01715
R097	AA15	GLU + H[+] + PRE ↔ AKG + CO2 + H2O + PHE	4.2.1.51	R01373
			2.6.1.1	R00694
			2.6.1.9	
R098	AA16	GLU + NAD + PRE ↔ AKG + CO2 + NADH + TYR	2.6.1.79	R07276
			1.3.1.78	R00733
R099	AA17	CHO + GLN ↔ ANTH + GLU + H[+] + PYR	4.1.3.27	R00986
R100	AA18	ANTH + H[+] + PRPP + SER ↔	2.4.2.18	R01073
		CO2 + GAP + PPi + TRYP + 2 H2O	5.3.1.24	R03509
			4.1.1.48	R03508
			4.2.1.20	R02722
R101	AA19	3 H2O + 2 NAD + ATP + GLN + PRPP →	2.4.2.17	R01071
		AICAR + AKG + HIS + Pi + 2 NADH + 2 PPi + 5 H[+]	3.6.1.31	R04035
			3.5.4.19	R04037
			5.3.1.16	R04640
			2.4.2.-	R04558
			/4.1.3.-	
			4.2.1.19	R03457
			2.6.1.9	R03243
			3.1.3.15	R03013
			1.1.1.23	R01158
				R01163
				R03012
R102	AA20	GLU + OXA ↔ AKG + ASP	2.6.1.1	R00355
R103	AA21	ASP + ATP + GLN + H2O → ADP + ASN + GLU + H[+] + Pi	6.3.5.4	R00578
R104	AA22	2 ATP + 2 H2O + CO2 + GLN →	6.3.5.5	R00575
		CaP + GLU + Pi + 2 ADP + 3 H[+]		
R105	AA23	2 GLU + ASP + ATP + CaP + NADH →	6.3.4.5	R01954
		AKG + AMP + ARG + FUM + H2O + NAD + PPi + Pi	4.3.2.1	R01086
R106	AA24	3 H[+] + 2 NADH + cGLU ↔ cPRO + 2 H2O + 2 NAD	1.2.1.41	R03313
			1.5.1.2	R01248
			2.7.2.11	R00239
R107	AA25	cAKG + O2 + cPRO ↔ CO2 + cHydPro + SUC	1.14.11.29	R03219
R108	AA26	ASP + ATP + H[+] + NADPH → ADP + ASA + NADP + Pi	2.7.2.4	R00480
			1.2.1.11	R02291

R109	AA27	1 H[+] + ASA + GLU + NADH + PYR ↔<br>AKG + CO2 + LYS + H2O + NAD	4.3.3.7<br>1.17.1.8<br>4.1.1.20<br>2.6.1.83<br>5.1.1.7	R10147<br>R04198<br>R00451<br>R07613<br>R02735
R110	AA28	ASA + H[+] + NADPH ↔ HSER + NADP	1.1.1.3	R01775
R111	AA29	ATP + H2O + HSER → ADP + H[+] + Pi + THR	2.7.1.39<br>4.2.3.1	R01771<br>R00996
R112	AA30	AcCoA + CYS + H2O + HSER ↔<br>Ace + CoA + HCYS + H[+] + NH4[+] + PYR	2.3.1.31<br>2.5.1.48<br>4.4.1.8	R01776<br>R03217<br>R01286
R113	AA31	HCYS + MTHF ↔ H[+] + MET + THF	2.1.1.13	R00946
R114	AA32	4.306 ATP + 3.306 H2O + 0.111 ALA + 0.094 GLY + 0.093 LEU + 0.059 cPRO + 0.059 VAL + 0.058 LYS + 0.057 THR + 0.055 SER + 0.05 cGLN + 0.05 cGLU + 0.047 ARG + 0.047 ASN + 0.047 ASP + 0.045 PHE + 0.036 ILE + 0.031 TYR + 0.022 MET + 0.017 HIS + 0.013 cHydPro + 0.012 CYS + 0.001 TRYP → PROTEIN + 4.306 ADP + 4.306 Pi + 4.314 H[+]		
R115	THF01	ATP + R5P → AMP + H[+] + PRPP	5.3.1.6<br>2.7.6.1	R01056<br>R01049
R116	THF02	5FTHF + H[+] ↔ H2O + MYLTHF	2.1.2.10	R02300
R117	THF03	H2O + MYLTHF ↔ H[+] + N10FTHF	3.5.4.9	R01655
R118	THF04	ATP + FORM + THF → ADP + N10FTHF + Pi	6.3.4.3	R00943
R119	THF05	MYLTHF + NADPH ↔ METHF + NADP	1.5.1.5	R01220
R120	THF06	H[+] + METHF + NADPH ↔ MTHF + NADP	1.5.1.20	R01224
R121	THF07	DHF + H[+] + NADPH ↔ NADP + THF	1.5.1.3	R00939
R122	PA01	ACP + AcCoA + H[+] ↔ AcACP + CoA	2.3.1.85<br>2.3.1.86	R01624
R123	PA02	ATP + AcCoA + CO2 + H2O → ADP + H[+] + MalCoA + Pi	6.4.1.2	R00742
R124	PA03	ACP + MalCoA ↔ CoA + MalACP	2.3.1.39	R01626
R125	PA04	10 H[+] + 10 NADPH + 5 MalACP + AcACP ↔<br>C12:0ACP + 5 ACP + 5 CO2 + 5 H2O + 10 NADP	2.3.1.179<br>1.1.1.100<br>4.2.1.59<br>1.3.1.9	R04963<br>R04964<br>R04965<br>R04724
R126	PA05	12 H[+] + 12 NADPH + 6 MalACP + AcACP ↔<br>C14:0ACP + 6 ACP + 6 CO2 + 6 H2O + 12 NADP	2.3.1.179<br>1.1.1.100<br>4.2.1.59<br>1.3.1.9	R04726<br>R04566<br>R04568<br>R04966
R127	PA06	14 H[+] + 14 NADPH + 7 MalACP + AcACP ↔<br>C16:0ACP + 7 ACP + 7 CO2 + 7 H2O + 14 NADP	2.3.1.179<br>1.1.1.100<br>4.2.1.59<br>1.3.1.9	R04968<br>R04543<br>R04544<br>R04969
R128	PA07	C16:0ACP + H[+] + NADH + O2 ↔<br>C16:1ACP + NAD + 2 H2O	1.14.19.11	R11108
R129	PA08	C16:1ACP + H[+] + NADH + O2 ↔<br>C16:2ACP + NAD + 2 H2O	1.14.19.23	
R130	PA09	C16:2ACP + H[+] + NADH + O2 ↔<br>C16:3ACP + NAD + 2 H2O		
R131	PA10	16 H[+] + 16 NADPH + 8 MalACP + AcACP ↔<br>C18:0ACP + 8 ACP + 8 CO2 + 8 H2O + 16 NADP	2.3.1.179<br>1.1.1.100<br>4.2.1.59<br>1.3.1.9	R07762<br>R07763<br>R10208<br>R07765
R132	PA11	C18:0ACP + H[+] + NADH + O2 ↔<br>C18:1ACP + NAD + 2 H2O	1.14.19.2	R03370
R133	PA12	C18:1ACP + H[+] + NADH + O2 ↔<br>C18:2ACP + NAD + 2 H2O	1.14.19.23	
R134	PA13	C18:2ACP + H[+] + NADH + O2 ↔<br>C18:3ACP + NAD + 2 H2O		

R135	PA14	GLYC3P + 0.474 C16:0ACP + 0.446 C18:3ACP + 0.276 C18:2ACP + 0.253 C16:3ACP + 0.16 C18:1ACP + 0.148 C16:2ACP + 0.104 C12:0ACP + 0.051 C14:0ACP + 0.048 C18:0ACP + 0.04 C16:1ACP ↔ PA + 2 ACP + 2 H[+]		
R136	PA15	H[+] + O2 + NADH + C16:3ACP → 2 H2O + NAD + C16:4ACP		
R137	TAG01	0.0186 C14:0ACP + 0.1275 C16:0ACP + 0.0387 C16:1ACP + 0.0137 C16:2ACP + 0.0191 C16:3ACP + 0.0019 C18:0ACP + 0.0753 C18:1ACP + 0.0967 C18:2ACP + 0.357 C18:3ACP + 0.2515 C16:4ACP → ACP + FA		
R138	TAG02	GLYC3P + FA → AG3P	2.3.1.15	R00851
R139	TAG03	AG3P + FA → DAG3P	2.3.1.51	R02241
R140	TAG04	H2O + DAG3P → DAG	3.1.3.4	R02239
			3.1.3.81	R09644
			2.7.1.107	R02240
R141	TAG05	DAG + FA → TAG	2.3.1.158	R05333
			2.3.1.20	R02251
			3.1.1.3	R01369
R142	Chl01	12 H[+] + 8 ATP + 8 cGLU + 8 NADPH + 2.5 O2 → cPPorphyrin + 4 NH4[+] + 6 cCO2 + 8 AMP + 8 NADP + 8 PPi + 13 H2O	6.1.1.17	R05578
			1.2.1.70	R04109
			5.4.3.8	R02272
			4.2.1.24	R00036
			2.5.1.61	R00084
			4.2.1.75	R03165
			4.1.1.37	R03197
			1.3.3.3	R03220
			1.3.3.4	R03222
R143	Chl02	18 H[+] + 15 NADPH + 8 ATP + 4 cGAP + 4 cPYR → cPhytyl-PP + 4 ADP + 4 AMP + 4 cCO2 + 7 PPi + 8 H2O + 15 NADP	2.2.1.7	R05636
			1.1.1.267	R05688
			2.7.7.60	R05633
			2.7.1.148	R05634
			4.6.1.12	R05637
			1.17.7.1	R08689
			1.17.7.3	R10859
			1.17.7.4	R05884
				R08210
			2.5.1.1	R01658
			2.5.1.10	R02003
			2.5.1.29	R02061
			1.3.1.83	R02063
R144	Chl03	ATP + H2O + MET → cAdMET + H[+] + PPi + Pi	2.5.1.6	R00177
R145	Chl04	cAdHCYS + H2O ↔cAd + HCYS	3.3.1.1	R00192
R146	Chl05	ATP + cAd → ADP + AMP + H[+]		
R147	Chl06	4 NADPH + 2.5 O2 + 2 ATP + cAdMET + Mg2[+] + cPPorphyrin + cPhytyl-PP → cAdHCYS + Chlorophyll + PPi + 2 ADP + 2 H2O + 2 Pi + 3 H[+] + 4 NADP	6.6.1.1	R03877
			2.1.1.11	R04237
			1.14.13.81	R06265
				R06266
				R06267
			1.3.1.75	R06272
				R06896
			1.3.1.33	R06286
				R03845
			2.5.1.62	R06284
				R09067
			1.14.13.122	R10080
			1.1.1.294	R08914
				R08915
				R09069
				R09070
			1.17.7.2	R09071

R148	NA01	4 ATP + 2 GLN + 2 H2O + ASP + CO2 + GLY + N10FTHF + PRPP → AICAR + FUM + PPi + THF + 2 GLU + 4 ADP + 4 Pi + 7 H[+]	2.4.2.17<br>3.6.1.31<br>5.3.1.16<br>2.4.2.-/<br>4.1.3.-	R01071<br>R04035<br>R04037<br>R04640<br>R04558
R149	NA02	ASP + CaP + H[+] + O2 + PRPP ↔ CO2 + H2O + H2O2 + PPi + Pi + UMP	2.4.2.10<br>4.1.1.23	R01870<br>R00965
R150	NA03	2 H2O2 ↔ O2 + 2 H2O	1.11.1.6	R00009
R151	NA04	ATP + UMP → ADP + UDP	2.7.4.22	R00158
R152	NA05	ATP + UDP ↔ ADP + UTP	2.7.4.6	R00156
R153	NA06	ATP + GLN + H2O + UTP → ADP + CTP + GLU + Pi + 2 H[+]	6.3.4.2	R00573
R154	NA07	ATP + CDP ↔ ADP + CTP	2.7.4.6	R00570
R155	NA08	AICAR + N10FTHF ↔ H2O + IMP + THF	2.1.2.3	R04560
R156	NA09	ATP + H2O + IMP + NAD + NH4[+] → AMP + GMP + NADH + PPi + 3 H[+]	1.1.1.205<br>6.3.5.2	R01130<br>R01230
R157	NA10	ATP + GMP → ADP + GDP	2.7.4.8	R00332
R158	NA11	ATP + GDP ↔ ADP + GTP	2.7.4.6	R00330
R159	NA12	ASP + IMP + GTP ↔ Pi + 2 H[+] + AMP + FUM + GDP		
R160	NA13	ATP + H[+] + METHF + NADPH + UDP → ADP + DHF + H2O + NADP + dTTP	1.17.4.1<br>2.7.4.9<br><br>2.1.1.45<br>2.7.4.6	R02018<br>R02098<br>R02094<br>R02101<br>R02093
R161	NA14	ATP + CDP + H[+] + NADPH → ADP + H2O + NADP + dCTP	1.17.4.1<br>2.7.4.6	R02024<br>R02326
R162	NA15	ATP + GDP + H[+] + NADPH → ADP + H2O + NADP + dGTP	1.17.4.1<br>2.7.4.6	R02019<br>R01857
R163	NA16	ATP + H[+] + NADPH ↔ H2O + NADP + dATP	2.7.4.6<br><br>1.17.4.1	R00124<br>R01137<br>R02017
R164	NA17	2.372 H2O + 1.372 ATP + 0.18 dATP + 0.18 dTTP + 0.32 dCTP + 0.32 dGTP → DNA + PPi + 1.372 ADP + 1.372 Pi + 2.372 H[+]	2.7.7.7	R00375<br>R00376<br>R00377<br>R00378
R165	NA18	1.4 H2O + 0.56 ATP + 0.34 GTP + 0.16 UTP + 0.34 CTP → 0.4 ADP + 0.4 H[+] + 0.4 Pi + PPi + RNA	2.7.7.6	R00435<br>R00441<br>R00442<br>R00443
R166	Car01	cGAP + cPYR → cCO2 + cDXP	2.2.1.7	R05636
R167	Car02	ATP + H[+] + 2 NADPH + CTP + cDXP ↔ 2 H2O + ADP + 2 NADP + PPi + CMP + cIPP	1.1.1.267<br>2.7.7.60<br>2.7.1.148<br>4.6.1.12<br>1.17.7.1<br>1.17.7.3<br>1.17.7.4	R05688<br>R05633<br>R05634<br>R05637<br>R08689<br>R10859<br>R05884
R168	Car03	ATP + H[+] + 2 NADPH + CTP + cDXP ↔ 2 H2O + ADP + 2 NADP + PPi + CMP + cDMAPP	1.1.1.267<br>2.7.7.60<br>2.7.1.148<br>4.6.1.12<br>1.17.7.1<br>1.17.7.3<br>1.17.7.4	R05688<br>R05633<br>R05634<br>R05637<br>R08689<br>R10859<br>R08210
R169	Car04	cIPP ↔ cDMAPP	5.3.3.2	R01123
R170	Car05	cDMAPP + 3 cIPP → 3 PPi + cGGPP	2.5.1.1<br>2.5.1.10<br>2.5.1.29	R01658<br>R02003<br>R02061

R171	Car06	2 cGGPP → 2 PPi + cPHYT	2.5.1.32	R10177
			2.5.1.99	R07916
R172	Car07	H2O + NAD + cPHYT → H[+] + NADH + cZCAR	1.3.5.5	R09652
				R09653
				R09654
R173	Car08	2 O2 + 2 NADH + cZCAR → 4 H2O + 2 NAD + cLYC	1.3.5.6	R07511
				R09656
				R09658
R174	Car09	cLYC → cBCAR	5.5.1.19	R05341
				R03824
R175	Car10	2 H[+] + 2 O2 + 2 NADPH + cBCAR → 2 H2O + 2 NADP + cZEA	1.14.13.129	R09747
R176	Car11	2 H[+] + 2 O2 + 2 NADH + cZEA ↔ 2 H2O + 2 NAD + cVIO	1.14.13.90	R10070
			1.23.5.1	
R177	Car12	cLYC → cACAR	5.5.1.18	R06963
			5.5.1.19	R06962
R178	Car13	cACAR → cLUT	1.14.13.129	R07530
			1.14.99.45	R07531
R179	Gly11	5.55 CARB → $CARB_P$		
R180	Glyc07	10.86 GLYC → $GLYC_P$		
R181	Gluc08	0.02 Starch → $Starch_P$		
R182	TAG06	1.06 TAG → TAG_P		
R183	Car14	1.86 BCAR → 1 $BCAR_P$		
R184	Chl07	1.11 Chlorophyll → 1 $Chlorophyll_P$		
R185	AA33	7.95 PROTEIN → $PROTEIN_P$		
R186	mu	32.687 ATP + 32.687 H2O + 1.432 PROTEIN + 3.258 GLYC + 0.666 CARB + 0.022 Chlorophyll + 0.061 DNA + 0.117 RNA + 0.560 PA → 1 BIOMASS + 32.687 H[+] + 32.687 ADP + 32.687 Pi		

No.	ID	Reaction	E.C.	KEGG ID
R187	Ox01	1.5 ADP + 1.5 H[+] + 1.5 Pi + FADH2 + 0.5 O2 → FAD + 1.5 ATP + 2.5 H2O		
R188	Ox02	3.5 H[+] + 2.5 ADP + 2.5 Pi + NADH + 0.5 O2 → NAD + 2.5 ATP + 3.5 H2O		
R189	Ox03	NAD + NADPH → NADH + NADP		
R190	Ox04	H2O + PPi → H[+] + 2 Pi		
R191	Ox05	AMP + ATP → 2 ADP		
R192	Ox06	ATP + H2O → ADP + H[+] + Pi		
R193	Ox07	1 NADH + 1 CMP → 1 NAD + 1 CDP		
R194	Ex01	Light_{ex} → Light		
R195	Ex02	CO2_{ex} ↔ CO2		
R196	Ex03	HCO3_{ex} ↔ HCO3		
R197	Ex04	O2 ↔ O2_{ex}		
R198	Ex05	SO4_{ex} → SO4		
R199	Ex06	NO3_{ex} → NO3		
R200	Ex07	NH4_{ex} → NH4		
R201	Ex08	Mg2_{ex} → Mg2[+]		
R202	Ex09	Pi_{ex} ↔ Pi		
R203	Ex10	H2O_{ex} ↔ H2O		
R204	T01	c3PG ↔ 3PG		
R205	T02	HCO3↔ cHCO3		
R206	T03	CO2 ↔ cCO2		
R207	T04	cAKG → AKG		
R208	T05	cASP ↔ ASP		
R209	T06	cDHAP → DHAP		
R210	T07	cGAP ↔ GAP		
R211	T08	cGLU ↔ GLU		
R212	T09	cGLN ↔ GLN		
R213	T10	cGLYCOL ↔ GLYCOL		
R214	T11	cGLYC3P → GLYC3P		
R215	T12	cGLYC → GLYC		
R216	T13	cNO2 ↔ NO2		
R217	T14	cPYR ↔ PYR		
R218	T15	cMAL ↔ MAL		
R219	T16	mAcCOA ↔ AcCoA		
R220	T17	mAKG ↔ AKG		
R221	T18	mMAL ↔ MAL		
R222	T19	mPYR ↔ PYR		

2 List of compounds

No.	Symbol	Name
C001	2OXOB	2-Oxobutanoate
C002	3PG	Glycerate-3-phosphate
C003	5FTHF	5-Formyl-THF
C004	6PG	6-Phosphogluconate
C005	AcACP	Acetyl-ACP
C006	AcCoA	Acetyl-CoA
C007	Ace	Acetate
C008	ACP	Acetyl-carrier protein
C009	ADP	Adenosine diphosphate
C010	AG3P	Acylglycerol-3-phosphate
C011	AICAR	5-Aminoimidazole-4-carboxamide ribonucleine
C012	AKG	2-Oxoglutarate (alpha-ketoglutarate)
C013	ALA	Alanine
C014	AMP	Adenosine monophosphate
C015	ANTH	Anthranilate
C016	ARG	Arginine
C017	ASA	L-Aspartic semialdehyde
C018	ASN	Asparagine
C019	ASP	Aspartate
C020	ATP	Adenosine triphosphate
C021	$BCAR_P$	Beta-Carotene (produced)
C022	BIOMASS	Biomass
C023	C12:0ACP	Dodecanoyl-ACP (Lauric acid)
C024	C14:0ACP	Tetradecanoyl-ACP (Myristic acid)
C025	C16:0ACP	Hexadecanoyl-ACP (Palmitic acid)
C026	C16:1ACP	Trans-Hexadec-2-enoyl-ACP (Palmitoleic acid)
C027	C16:2ACP	Hexadecadienoic acid
C028	C16:3ACP	Hexadecatrienoic acid
C029	C16:4ACP	Hexadecatetraenoic acid
C030	C18:0ACP	Octadecanoyl-ACP (Stearic acid)
C031	C18:1ACP	Cis-11-ocadecanoate-ACP (Oleic acid)
C032	C18:2ACP	Linoleic acid
C033	C18:3ACP	Alpha-linoleic acid
C034	c3PG	Glycerate-3-phosphate (Chloroplast)
C035	c6PG	6-Phosphogluconate (Chloroplast)
C036	cACAR	Alpha-Carotene (Chloroplast)
C037	cAd	Adenosine (Chloroplast)
C038	cAdHCYS	S-Adenosyl-L-homocysteine (Chloroplast)
C039	cAdMET	S-Adenosyl-L-methionine (Chloroplast)
C040	cADP-G	ADP-Glucose (Chloroplast)
C041	cAKG	2-Oxoglutarate (Chloroplast)
C042	CaP	Carbamoyl phosphate
C043	cAPS	Adenylyl sulfate (Chloroplast)
C044	CARB	Carbohydrate
C045	$CARB_P$	Carbohydrate (produced)
C046	cASP	Aspartate (Chloroplast)
C047	cBCAR	Beta-Carotene (Chloroplast)
C048	cCO2	Carbon dioxide (Chloroplast)
C049	cDHAP	Dihydroxyacetone-P (Chloroplast)
C050	cDMAPP	Dimethylallyl-pyrophosphate (Chloroplast)
C051	CDP	Cytidine diphosphate (Chloroplast)
C052	cDXP	Deoxy-xylulose 5-phosphate (Chloroplast)
C053	cE4P	Erythrose 4-phosphate (Chloroplast)
C054	cF16P	Fructose 1,6-bisphosphate (Chloroplast)
C055	cF6P	Fructose 6-phosphate (Chloroplast)
C056	cG1P	Glucose 1-phosphate (Chloroplast)
C057	cG6P	Glucose 6-phosphate (Chloroplast)

C058	cGAP	Glyceraldehyde 3-phosphate (Chloroplast)
C059	cGGPP	Geranylgeranyl-pyrophosphate (Chloroplast)
C060	cGLN	Glutamine (Chloroplast)
C061	cGLU	Glutamate (Chloroplast)
C062	cGLYC	Glycerol (Chloroplast)
C063	cGLYC3P	Glycerol 3-phosphate (Chloroplast)
C064	cGLYCOL	Glycolate (Chloroplast)
C065	cGLYCOL2P	Glycolate-2P (Chloroplast)
C066	cH2S	Hydrogen sulfide (Chloroplast)
C067	cHCO3[-]	Bicarbonate (Chloroplast)
C068	Chlorophyll	Chlorophyll
C069	$\text{Chlorophyll}_{\text{P}}$	Chlorophyll (produced)
C070	CHO	Chorismate
C071	cHydPro	Hydroxyproline (Chloroplast)
C072	cIPP	Isopentyl-pyrophosphate (Chloroplast)
C073	cLUT	Lutein (Chloroplast)
C074	cLYC	Lycopene (Chloroplast)
C075	cMAL	Malate (Chloroplast)
C076	CMP	Cytidine monophosphate
C077	cNO2	Nitrite (Chloroplast)
C078	CIT	Citrate
C079	CO2	Carbon dioxide
C080	CO2_{ex}	Carbon dioxide (extracellular)
C081	CoA	Coenzyme A
C082	cOXA	Oxaloacetate (Chloroplast)
C083	cPHYT	Phytoene (Chloroplast)
C084	cPhytyl-PP	Phytyl-diphosphate (Chloroplast)
C085	cPPorphyrin	Protoporphyrine (Chloroplast)
C086	cPRO	Proline (Chloroplast)
C087	cPYR	Pyruvate (Chloroplast)
C088	cR5P	Ribose 5-phosphate (Chloroplast)
C089	cRu15DP	Ribulose 1,5-bisphosphate (Chloroplast)
C090	cRu5P	Ribulose 5-phosphate (Chloroplast)
C091	cS7P	Sedoheptulose 7-phosphate (Chloroplast)
C092	cSO3	Sulphite (Chloroplast)
C093	cSO4	Sulphate (Chloroplast)
C094	CTP	Cytidine triphosphate
C095	cVIO	Violaxanthin (Chloroplast)
C096	cX5P	Xylulose 5-phosphate (Chloroplast)
C097	CYS	Cysteine
C098	cZCAR	Zeta-carotene (Chloroplast)
C099	cZEA	Zeaxanthin (Chloroplast)
C100	DAG	Diacylglycerol
C101	DAG3P	Diacylglycerol-3-phosphate
C102	dATP	Deoxy ATP
C103	dCTP	Deoxy CTP
C104	dGTP	Deoxy GTP
C105	DHA	Dihydroxyacetone (Glycerone)
C106	DHAP	Dihydroxyacetone-P
C107	DHF	Dihydrofolate
C108	DNA	Deoxyribonucleic acid
C109	dTTP	Deoxy TTP
C110	E4P	Erythrose 4-phosphate
C111	F16P	Fructose 1,6-bisphosphate
C112	F6P	Fructose 6-phosphate
C113	FA	Fatty acids
C114	FAD	Flavin adenine dinucleotide oxidized
C115	FADH2	Flavin adenine dinucleotide reduced
C116	FORM	Formic acid
C117	FUM	Fumarate
C118	G1P	Glucose 1-phosphate

C119	G6P	Glucose 6-phosphate
C120	GA	Glyceraldehyde
C121	GAP	Glyceraldehyde 3-phosphate
C122	GDP	Guanosine diphosphate
C123	GLN	Glutamine
C124	GLU	Glutamate
C125	GLUC	Glucose
C126	GLY	Glycine
C127	GLYC	Glycerol
C128	GLYC3P	Glycerol 3-phosphate
C129	GLYCA	Glycerate
C130	$GLYC_P$	Glycerol (produced)
C131	GLYCOL	Glycolate
C132	GLYOX	Glyoxylate
C133	GMP	Guanosine monophosphate
C134	GTP	Guanosine triphosphate
C135	H2O	Water
C136	H2O2	Hydrogen peroxide
C137	$H2O_{ex}$	Water (extracellular)
C138	HCO3[-]	Bicarbonate
C139	$HCO3_{ex}$	Bicarbonate (extracellular)
C140	HCYS	Homocysteine
C141	HIS	Histidine
C142	HSER	Homoserine
C143	HydPyr	3-Hydroxypyruvate
C144	ILE	Isoleucine
C145	IMP	Inosine monophosphate
C146	LEU	Leucine
C147	Light	Photons
C148	$Light_{ex}$	Photons (extracellular)
C149	LYS	Lysine
C150	mAcCoA	Acetyl-CoA (Mitochondrium)
C151	mAKG	2-Oxoglutarate (Mitochondrium)
C152	MAL	Malate
C153	MalACP	Malonyl-ACP
C154	MalCoA	Malonyl-CoA
C155	mCIT	Citrate (Mitochondrium)
C156	mCO2	Carbon dioxide (Mitochondrium)
C157	MET	Methionine
C158	METHF	5,10-Methylene-THF
C159	mFUM	Fumarate (Mitochondrium)
C160	Mg2[+]	Magnesium
C161	$Mg2[+]_{ex}$	Magnesium (extracellular)
C162	mHCO3[-]	Bicarbonate (Mitochondrium)
C163	mMAL	Malate
C164	mOXA	Oxaloacetate
C165	mPYR	Pyruvate
C166	mSUC	Succinate
C167	mSUCCoA	Succinyl Coenzyme A
C168	MTHF	Methyl-THF
C169	MYLTHF	5,10-Methenyl-THF
C170	N10FTHF	10-Formyl-THF
C171	NAD	Nicotinamide oxidized
C172	NADH	Nicotinamide reduced
C173	NADP	Nicotinamidephosphate oxidized
C174	NADPH	Nicotinamidephosphate reduced
C175	NH4[+]	Ammonium
C176	$NH4[+]_{ex}$	Ammonium (extracellular)
C177	NO2	Nitrite
C178	NO3	Nitrate
C179	$NO3_{ex}$	Nitrate (extracellular)

C180	O2	Oxygen
C181	$O2_{ex}$	Oxygen (extracellular)
C182	OXA	Oxaloacetate
C183	PA	Phosphatidic acid
C184	PEP	Phosphoenolpyruvate
C185	PHE	Phenylalanine
C186	Pi	Orthophosphate
C187	Pi_{ex}	Orthophosphate (extracellular)
C188	PPi	Pyrophosphate
C189	PRE	Prephanate
C190	PROTEIN	Protein
C191	$PROTEIN_{P}$	Protein (produced)
C192	PRPP	Phosphorybosylpyrophosphate
C193	PYR	Pyruvate
C194	R5P	Ribose 5-phosphate
C195	RNA	Ribonucleic acid
C196	RU5P	Ribulose 5-phosphate
C197	S7P	Sedoheptulose 7-phosphate
C198	SER	Serine
C199	$SO4_{ex}$	Sulphate (extracellular)
C200	STARCH	Starch
C201	$STARCH_{P}$	Starch (produced)
C202	SUC	Succinate
C203	TAG	Triacylglycerol
C204	TAG_{P}	Triacylglycerol (produced)
C205	THF	Tetrahydrofolate
C206	THR	Threonine
C207	TRYP	Tryptophan
C208	TYR	Tyrosine
C209	UDP	Uridine diphosphate
C210	UMP	Uridine monophosphate
C211	UTP	Uridine triphosphate
C212	VAL	Valine
C213	X5P	Xylulose 5-phosphate

3 Composition of biomacromolecules

Protein

Amino acid	Molar mass AA (g mol^{-1})	Molar fraction (mol AA mol^{-1} Protein)
Alanine	89.09	11.07
Arginine	174.20	4.67
Asparagine	132.12	4.68
Aspartate	133.10	4.68
Cysteine	121.16	1.17
Glutamine	146.15	4.96
Glutamate	147.13	4.96
Glycine	75.07	9.36
Histidine	155.16	1.69
Hydroxyproline	131.13	1.30
Isoleucine	131.17	3.59
Leucine	131.17	9.25
Lysine	146.19	5.78
Methionine	149.21	2.22
Phenylalanine	165.19	4.45
Proline	115.13	5.92
Serine	105.09	5.51
Threonine	119.12	5.71
Tryptophan	204.23	0.10
Tyrosine	181.19	3.08
Valine	117.15	5.86
Av. molar mass	125.74	g mol^{-1} Protein

Fatty acids

Fatty acid	Fraction of PA	Fraction on neutral lipids	Molar mass (g mol^{-1})	Molar mass PA (g mol^{-1})	Molar mass FA (g mol^{-1})
C12:0	0.05	0.000	200	10.00	0
C14:0	0.03	0.016	228	6.84	3.65
C16:0	0.24	0.123	256	61.44	31.49
C18:0	0.05	0.002	274	13.70	0.55
C16:1	0.02	0.037	254	5.08	9.40
C16:2	0.07	0.013	252	17.64	3.28
C16:3	0.13	0.018	250	32.50	4.5
C18:1	0.08	0.080	318	25.44	25.44
C18:2	0.14	0.102	316	44.24	32.23
C18:3	0.22	0.373	314	69.08	117.12
C16:4	0.00	0.235	248	0.00	58.28
Av. molar mass PA	571.92	g mol^{-1} PA			
Av. molar mass FA	946.80	g mol^{-1} FA			

The biomacromolecule content was calculated as follows:

$$\text{Biomacromolecule content (mmol g}^{-1}\text{ dw)} = \frac{\text{Cellular content of biomacromolecule (g g}^{-1}\text{ dw)}}{\text{Molecular weight of biomacromolecule (g mol}^{-1}\text{)}} \cdot 1000 \text{ mmol mol}^{-1}$$

Biomass composition

		LL<br>Scenario I - green cell state		HL-ND<br>Scenario II - orange cell state		Minimal functional<br>biomass composition
Compound	Molar mass<br>(g mol^{-1})	Fraction<br>(%)	Composition<br>(mmol g^{-1} dw)	Fraction<br>(%)	Composition<br>(mmol g^{-1} dw)	normalized to 1<br>(mmol g^{-1} dw)
Functional biomass						
Protein	125.73	44	3.50	9	0.72	1.432
Glycerol	92.09	15	1.63	15	1.63	3.258
Carbohydrates	180.16	14	0.78	6	0.33	0.666
Chlorophyll	900.49	1	0.01	1	0.01	0.022
DNA	326.11	1	0.03	1	0.03	0.061
RNA	340.80	2	0.06	2	0.06	0.117
Phospholipids	571.92	16	0.28	16	0.28	0.560
Storage compounds						
β-carotene	536.89	1	0.02	10	0.19	
TAG	946.80	1	0.01	10	0.11	
Starch	48648	5	0.00	30	0.01	
Sum functional		93		50		
Sum storage		7		50		
total		100		100		

The energy requirements for the formation of protein, DNA, RNA, and chlorophyll were assumed to be 4.306, 1.372, 0.4, and 2.0 mol ATP per mol of the respective macromolecule (Kliphuis, A.M.J., Klok, A.J., Martens, D.E. et al. J Appl Phycol (2012) 24: 253. https://doi.org/10.1007/s10811-011-9674-3).
